# OCT parameters in patients with diabetic maculopathy

**Published:** 2014

**Authors:** R Serban, M Cioboata, C Chiotan, C Cornăcel, R Liora, A Anghelie

**Affiliations:** * Ophthalmology Emergency Hospital Bucharest; ** Department of HIV / AIDS, Office of Ophthalmology, ”Prof. Dr. Matei Bals” Institute of Infectious Diseases, Bucharest

**Keywords:** clinically significant macular edema, triamcinolone acetonide, bevacizumab, optical coherence tomography

## Abstract

The treatment of the clinically significant macular edema in patients with diabetic maculopathy by the intravitreal administration of corticosteroids and antiangiogenic factors requires rapid and more accurate investigation methods aimed at following up the dynamic evolution of the structural parameters of the retina.

**The purpose** of the study is to compare the outcome of OCT parameters for each group (Group A treated by intravitreal administered triamcinolone acetonide and Group B treated by intravitreal administered triamcinolone acetonide in combination with bevacizumab).

**Methods** involved the selection of cases, dynamic measurements of the retina structures, so that, at the end, the data from the two groups of patients could be compared.

The results showed a positive development in the short and medium term in both groups of patients.

**Abbreviations:** OCT = optical coherence tomography, WESDR = epidemiological study of diabetic retinopathy Wisconsin, ETDRS = study early treatment of diabetic retinopathy, retinal thickness GRC = central-VMT total macular volume, VEGF, vascular endothelial growth factor

## Introduction

Macular edema represents one of the main reasons that lead to decreased visual acuity in patients diagnosed with diabetic retinopathy. Macular edema is defined as the retinal thickening by the pathological accumulation of extracellular fluid in the macular area [**4**]. Histologically, edema fluid is present in the outer plexiform layer and the internal nuclear layer [**7**].

Edema incidence increases with the duration and severity of diabetes and is more common in type II diabetes mellitus [**4**,**5**].

The incidence of macular edema in WESDR after 10 years of development is of 20.1% in patients with type I diabetes mellitus, of 25.4% in patients with type II diabetes insulin dependent and 13.9% in patients with type II diabetes mellitus without insulin requirements [**7**].

This study showed that the incidence of macular edema also increases with the severity of retinopathy in both type I and type II diabetes [**7**].

Diabetic macular edema is often associated with hard exudates, intraretinal hemorrhages and microaneurysms, which usually represent the extracellular fluid source [**6**].

Long lasting intraretinal fluid can accumulate in parafoveal cystic spaces forming cystoid macular edema [**5**].

The ETDRS defined the clinically significant macular edema as one of the following conditions (grading in order of severity):

I. retinal edema localized at less than 500 micrometers from the center of the macula;

II. hard exudates at less than 500 micrometers from the center of the macula, if they are associated with adjacent macular thickening;

III. at least one papillary diameter retinal thickening, located at less than a papillary diameter from the center of the macula [**7**].

The clinical diagnosis of macular edema involves biomicroscopy fundus examination by using either Goldman lens or Volk lens and stereoscopic fundus photography. These clinical diagnostic methods are subjective in assessing the present and the extent of edema.

The last decade has brought significant achievements in terms of ocular imaging allowing the objectification of retinal edema.

The optical coherence tomography (Ocular Coherence Tomography OCT) seems to be the most sensitive and reliable method for the thickness measurement of the edematous retina. It also shows the internal structure of the retina, the presence of the intraretinal cystic spaces, and the presence of a thickened hialoide or vitreoretinal traction syndrome.

The following parameters are represented by the mean central retinal thickness (MCRT) and total macular volume (TMV). MCRT is expressed in micrometers and TMV in mm [**3**].

Regarding the therapeutic possibilities in well-selected cases, clinically significant macular edema treatment involves an intravitreal injection of corticosteroids and antiangiogenic factors.

Glucocorticoids are used to treat diabetic macular edema for their anti-inflammatory effect, which can antagonize at least a part of the pathological processes involved in the occurrence of edema [**1**]. Glucocorticoids stabilize the capillary wall with consequent improvement of endothelial barrier function [**1**].

This process primarily occurs by the modulation of the synthesis and the expression at the site of endothelial cells, of molecules belonging to the class of intercellular tight junctions (occludin proteins) [**2**]. Secondly, glucocorticoids inhibit leukocyte adhesion to the endothelium, inhibiting further local inflammation [**2**]. On the other hand, the anti-inflammatory effect is due to the local inhibition of VEGF (vascular endothelial growth factor) synthesis also known as the factor that increases capillary permeability [**2**].

Glucocorticoid triamcinolone acetonide seems to be most appropriate in this aspect. It is presented in vials as a white crystalline suspension, 40 mg triamcinolone in 1 ml solution (e.g. Vitreal S, very frequently used).

VEGF (vascular endothelial growth factor) was first discovered as a factor that increases vascular permeability and even called “vascular permeability factor”. The secretion of VEGF in the retina can cause increased capillary permeability and barrier damage. For this reason, it is considered that VEGF plays an important role in diabetic macular edema. Furthermore, the concentration of vitreal VEGF correlates with the severity of the macular edema [**3**].

AntiVEGF agents are representations of pegaptanib sodium, bevacizumab and ranibizumab, listed in the chronological order of their occurrence. Bevacizumab is the most commonly used antiVEGF agent [**3**].

Bevacizumab (e.g. Avastin, Genentech) is an anti-VEGF monoclonal antibody approved by the FDA in February 2004 for intravenous metastatic colorectal cancer. Intravitreal injection is used off-label and bevacizumab seems as effective as ranibizumab, but is considerably less expensive. 

## Material and methods

The present study followed up patients diagnosed with clinically significant macular edema and treated with intravitreal injection of triamcinolone acetonide or triamcinolone acetonide in combination with bevacizumab, for a period of at least six months.

The patients’ evolution was followed in terms of possible post intervention complications, functional parameters (visual acuity) and OCT parameters of macular region (mean retinal thickness in the center of the fovea - MCRT, total macular volume - TMV).

Thus, based on selectivity, 295 patients (460 eyes) divided into 3 groups were selected it as follows:

- Group A: 90 patients (161 eyes) treated with intravitreal injection of triamcinolone acetonide;

- Group B: 112 patients (180 eyes) treated with intravitreal injection of triamcinolone acetonide and bevacizumab;

- Group C: 93 patients (119 eyes), representing the control group.

Patients were randomly assigned for each group.

The OCT parameters were used to assess the retinal morphostructural dynamic evolution.

All the patients were investigated by using the same device and measurements performed by the same person and under the same conditions.

Measurements were performed at baseline, following that in a range of at least six months investigations to be performed after each intervention (intravitreal injection), or by routine. It should be mentioned that the last OCT measurement was performed at least one month after the intravitreal injection.

Most patients were subjected to three interventions within less than one month.

## Results

1. Group A OCT parameters.

**Table 1** shows OCT parameters at the time of enrollment, during treatment, and at least one month after the last intervention, as well as the standard deviation.

**Table 1:** Group A OCT parameters

OCT parameters	Average ± Standard deviation - μm
Initial MCRT	468,29 ± 133,49
Intermediate MCRT	402,22 ± 131,17
Final MCRT	371,84 ± 121,19
Initial TMV	9,97 ± 2,15
Intermediate TMV	9,26 ± 2,09
Final TMV	9,01 ± 2,04

The decrease of mean values following triamcinolone administration to time 0 meant reducing macular edema.

OCT parameters analysis showed that the differences observed between the initial and final measurements were statistically significant (p < 0.05), as shown in **Table 2**.

**Table 2:** OCT parameters range (Group A)

	OCT parameters range
	(average variation ± standard deviation)- μm
Initial MCRT – final MCRT	96,45 ± 121,33
Initial TMV – final TMV	0,96 ± 2,11

As a result, intravitreal triamcinolone administration had an obvious effect in reducing diabetic macular edema.

2. Group B OCT parameters.

Similar measurements were found in group B in patients treated with intravitreal administration of triamcinolone acetonide and bevacizumab (**Table 3**).

**Table 3:** B group OCT parameters

OCT parameters	Average ± Standard deviation - μm
Initial MCRT	446,33 ± 114,73
Intermediate MCRT	367,06 ± 91,17
Final MCRT	328,06 ± 85,87
Initial TMV	10,11 ± 2,21
Intermediate TMV	9,41 ± 2,03
Final TMV	9,12 ± 2,09

The differences observed between the initial and final measurements are statistically significant (p < 0.05), **Table 4**.

**Table 4:** OCT parameters range (group B)

	OCT parameters range
	(average variation ± standard deviation)- μm
Initial MCRT – final MCRT	118,26 ± 107,56
Initial TMV – final TMV	0,99 ± 2,05

3. C group OCT parameters.

An increase of the retinal edema with the progression of diabetic retinopathy was observed in the control group, six months from the start of the monitoring, **Table 5**.

**Table 5:** Group C OCT parameters

OCT parameters	Average ± Standard deviation - μm
MCRT at 3 months	433,21 ± 112,67
MCRT at 6 months	452,81 ± 113,02
TMV at 3 months	10,05 ± 2,14
TMV at 6 months	10,39 ± 2,19

## Conclusions

The OCT measurements (MCRT and TMV) recorded a decrease in macular edema in both groups (A and B), in the interval between initiation of therapy and at least one month after the last intravitreal injection.

What should be mentioned is that all the imaging investigations were performed with the same device and under the same conditions.

For group B (triamcinolone administration + bevacizumab) MCRT average decrease was of 22.61% compared with that in group A of patients. The average TMV decreased with 3.12% more than in the group who received triamcinolone and bevacizumab.

The average retinal thickness at 6 months after the first triamcinolone injection was lower than the baseline value.

For group B, the reduction in macular edema measured by the two parameters was significantly lower at 6 months compared to baseline, demonstrating that the administrations’ combined effectiveness in diabetic macular edema is the same as in the administration of triamcinolone.

Thus, it can be concluded that there is a favorable outcome for short and medium term in both groups of patients.
